# Physiological changes after fluid bolus therapy in sepsis: a systematic review of the contemporary literature

**DOI:** 10.1186/cc14037

**Published:** 2014-12-03

**Authors:** NJ Glassford, GM Eastwood, R Bellomo

**Affiliations:** 1 Department of Intensive Care, Austin Hospital, Heidelberg, VIC, Australia; 2 ANZICS Research Centre, School of Public Health and Preventative Medicine, Monash University, Melbourne, VIC, Australia; 3 School of Nursing and Midwifery, Faculty of Health, Deakin University, Burwood, VIC, Australia

## Introduction

Fluid bolus therapy (FBT) is a ubiquitous intervention in intensive care. However, the physiological effects in the critically ill are poorly understood. Therefore, we systematically reviewed the contemporary literature to determine the current practice and effect of FBT in the management of severe sepsis and septic shock.

## Methods

We interrogated the MEDLINE, CENTRAL and EMBASE electronic reference databases using a combination of terms to define a set of records of studies of fluid administration in patients with severe sepsis or septic shock. To achieve contemporary relevance, results were limited to English-language studies in adults between 2010 and 2013.

## Results

**Figure 1:**
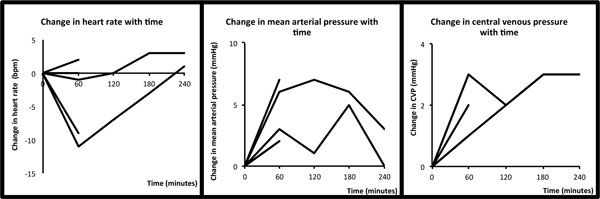
**Haemodynamic changes following FBT at 1, 2, 3 and 4 hours**.

We identified 22 prospective observational studies, four retrospective observational studies, two quasi-experimental studies, and five randomised controlled trials (RCTs), 41 boluses in total. No RCT compared FBT with alternative interventions. The median fluid bolus was 500 ml (range: 100 to 1,000 ml) administered over 30 minutes (range: 10 to 60 minutes) and 0.9% sodium chloride solution was the most commonly administered. Although 17 studies describe the temporal course of physiological changes after FBT in 31 patient groups, only three studies describe the physiological changes at 60 minutes, and only one study beyond this point (Figure 1). No studies related the physiological changes after FBT with clinically relevant outcomes.

## Conclusion

There is a need for obtaining randomised controlled evidence for the physiological effects of FBT in patients with severe sepsis and septic shock beyond the period immediately following its administration.

